# Decision-Tree, Rule-Based, and Random Forest Classification of High-Resolution Multispectral Imagery for Wetland Mapping and Inventory

**DOI:** 10.3390/rs10040580

**Published:** 2018

**Authors:** Tedros M. Berhane, Charles R. Lane, Qiusheng Wu, Bradley C. Autrey, Oleg A. Anenkhonov, Victor V. Chepinoga, Hongxing Liu

**Affiliations:** 1 Pegasus Technical Services, Inc., C/O U.S. Environmental Protection Agency, Cincinnati, OH 45219, USA; berhane.tedros@epa.gov; 2 Office of Research and Development, U.S. Environmental Protection Agency, Cincinnati, OH 45268, USA; autrey.brad@epa.gov; 3 Department of Geography, Binghamton University, State University of New York, Binghamton, NY 13902, USA; wqs@binghamton.edu; 4 Laboratory of Floristics and Geobotany, Institute of General and Experimental Biology SB RAS, 670047 Ulan-Ude, Russia; anen@yandex.ru; 5 Laboratory of Physical Geography and Biogeography, V.B. Sochava Institute of Geography SB RAS, 664033 Irkutsk, Russia; victor.chepinoga@gmail.com; 6 Department of Botany, Irkutsk State University, 664003 Irkutsk, Russia; 7 Department of Geography, University of Cincinnati, Cincinnati, OH 45220, USA; hongxing.liu@uc.edu

**Keywords:** freshwater wetland, Lake Baikal, methodological comparison, Selenga River Delta, WorldView-2

## Abstract

Efforts are increasingly being made to classify the world’s wetland resources, an important ecosystem and habitat that is diminishing in abundance. There are multiple remote sensing classification methods, including a suite of nonparametric classifiers such as decision-tree (DT), rule-based (RB), and random forest (RF). High-resolution satellite imagery can provide more specificity to the classified end product, and ancillary data layers such as the Normalized Difference Vegetation Index, and hydrogeomorphic layers such as distance-to-a-stream can be coupled to improve overall accuracy (OA) in wetland studies. In this paper, we contrast three nonparametric machine-learning algorithms (DT, RB, and RF) using a large field-based dataset (*n* = 228) from the Selenga River Delta of Lake Baikal, Russia. We also explore the use of ancillary data layers selected to improve OA, with a goal of providing end users with a recommended classifier to use and the most parsimonious suite of input parameters for classifying wetland-dominated landscapes. Though all classifiers appeared suitable, the RF classification outperformed both the DT and RB methods, achieving OA >81%. Including a texture metric (homogeneity) substantially improved the classification OA. However, including vegetation/soil/water metrics (based on WorldView-2 band combinations), hydrogeomorphic data layers, and elevation data layers to increase the descriptive content of the input parameters surprisingly did not markedly improve the OA. We conclude that, in most cases, RF should be the classifier of choice. The potential exception to this recommendation is under the circumstance where the end user requires narrative rules to best manage his or her resource. Though not useful in this study, continuously increasing satellite imagery resolution and band availability suggests the inclusion of ancillary contextual data layers such as soil metrics or elevation data, the granularity of which may define its utility in subsequent wetland classifications.

## 1. Introduction

Wetlands are dynamic environments existing at the terrestrial-aquatic interface. As such, they are vulnerable to a wide range of human-mediated environmental and hydrological alterations associated with population growth, urbanization, and increased human development activities. Global and regional climate change, particularly temperature alterations and changing precipitation trends, have considerably affected wetland ecosystems [1,2]. Despite their vital functions in biodiversity and ecosystem services (e.g., [3]), wetlands have experienced extensive losses throughout the world in recent decades [4–8]. Intelligent planning measures and effective management policies need to be formulated to conserve and protect existing wetland resources; to mitigate negative anthropogenic impacts on wetlands; and to maintain wetland integrity, functioning, and resilience. Wetland mapping and inventory are critical to acquiring the scientific knowledge about wetland habitats, including their location, extent, and spatial distribution, as well as their vegetation composition, structure, and density. Once this knowledge is gained, effective management can ensue.

Satellite data have long been utilized to augment and supplant field- and aerial-based assessment techniques [9,10]. However, due to their high spatial heterogeneity and temporal hydrologic variability, wetlands have been among the most difficult ecosystems to classify with remotely sensed imagery [9,11–14]. In past decades, automated image classification approaches were extensively adopted to process satellite remote sensing imagery for mapping and studying wetlands at a large spatial scale, reducing inconsistencies associated with human interpretation, and creating reproducible wetland maps [15–17]. Satellite data require unsupervised or supervised classification of the spectral signatures for wetland characterization, and classification approaches have advanced in concert with satellite advancements [9,18–27]. There is now a wealth of classifiers, including Iterative Self-Organizing Data Analysis Technique (ISODATA) [28–30], maximum likelihood [30,31], artificial neural network [32,33], support vector machine [34], and ensemble approaches [35].

Three increasingly employed approaches for classifying remote sensing images are decision-tree (DT), rule-based (RB), and random forest (RF) classification. All three methods are nonparametric, and as such they are not constrained by the distribution of the predictor variables. The DT method is an efficient inductive machine learning technique [36–38]. A DT consists of a root-nodes-branches-leaf flowchart that is created to effectively bin data by recursively testing attributes of the dataset at each tree node, with branches representing the different outcomes leading to subsequent nodes, until a leaf (or terminal node) is created, representing a class. Compared with traditional classification methods such as the maximum likelihood and linear discriminant function classifiers, the DT method has a number of advantages [39,40]. As a nonparametric classifier, it is robust with respect to nonlinear interactions between variables and relatively insensitive to noisy relationships between input attributes and class labels [41]. It makes no assumptions regarding normality for the predictor variables and can easily accommodate both continuous and categorical data from various measurement scales (i.e., nominal, ordinal, interval, and ratio scales [36–38]). Examples of wetland classifications using DTs from the literature include Baker et al. [42], who used a DT-based classification method with Landsat Enhanced Thematic Mapper Plus imagery and both topographic and soil data to distinguish wetlands and riparian areas with 86% overall accuracy. Similarly, Wright and Gallant [13] used DTs to combine Landsat Thematic Mapper imagery and ancillary environmental data to discriminate among five palustrine wetland types in a large national park in the western US.

The RB approach creates a series of “if-then” rules to effectively classify landscapes, and can similarly couple different types of data in the process (e.g., [39,43]). The RB approach is similar to the DT approach, but generally has fewer rules and contains contextual information within the ruleset, hence it is simpler to understand than the complex bifurcating DTs. Domain knowledge, spatial context, and associations can also be integrated into the RB classification algorithm. For instance, Li and Chen [44] used Landsat ETM+, Radarsat Synthetic Aperture Radar (SAR), and elevation data in a series of “if-then” rules to classify each pixel in their study area as open bog, open fen, tree bog, marsh, or swamp, with classification accuracy ranging from 71 to 92%. Sader et al. [45] applied RB methods to effectively classify Landsat TM images for discrimination between forested wetlands and uplands. Houhoulis and Michener [46] created an RB approach to detect wetland change coupling SPOT-XS imagery and aerially derived wetland inventory data.

The RF approach is a relatively novel classification technique based on ensemble machine learning and has been increasingly used as a classifier of choice for remote sensing of different types of wetlands and aquatic habitats (e.g., [47]). The RF approach, like DT and RB, is nonparametric, robust to normal distribution departures, and can be used for both classifications and regressions, as well as determining variable importance [48,49]. Thus, RF has many of the benefits of DT and RB classification while overcoming several limitations, such as overfitting [50]. Errors of bias and variance can affect the DT and RB approaches, whereas RF avoids both errors through random selection of input-predictor variables and use of different subsets of the same training dataset [50,51]. Examples of RF used in wetland classification include Tian et al. [52], who used fused Pleiade-1B and multitemporal Landsat-8 data for mapping wetland cover in an arid region in China. Corcoranet al. [53] used multisource and multitemporal remote sensing and ancillary information such as radar and optical data, topographic data, and soil characteristics for mapping mixed managed and natural wetlands of woody and herbaceous plants in Minnesota, USA. Van Beijma et al. [54] used both the S-band and X-band quad-polarimetric airborne SAR, elevation data, and optical remotely sensed data for mapping natural coastal salt marsh vegetation habitats. Disadvantages of RF include a relatively longer processing time and model complexity, especially in comparison with the DT and RB methods [51].

Increased interest in wetland ecosystems has resulted in marked advances in characterizing and classifying wetland structure (frequently controlled by inundation patterning; see [55,56]). An abundance of advanced satellite platforms and spatial data coupled with an understanding of differences in functional rates based on wetland typologies (e.g., [57,58]) portends a need to choose effective classification techniques. Which technique is chosen will hinge on the needs of the end user, who could consider trade-offs such as the simplicity of DT or contextual nature of RB versus the classification robustness of the higher-complexity RF approach. In this paper, we contrast and report the efficacy and accuracy of three different classifiers, the DT, RB, and RF approaches, using a large wetland vegetation dataset and high-resolution imagery. As end users might have different resources available, we further explore changes in overall accuracy (OA) using parsimonious inputs (e.g., a spartan four-band analysis) as well as highly parameterized inputs (e.g., eight bands plus spectral metrics, hydrogeomorphic variables, and elevation data). The goal of this study is to provide methodological recommendations to effectively, efficiently, and robustly classify a given wetland landscape.

## 2. Methods

### 2.1. Study Area

**Figure 1.**
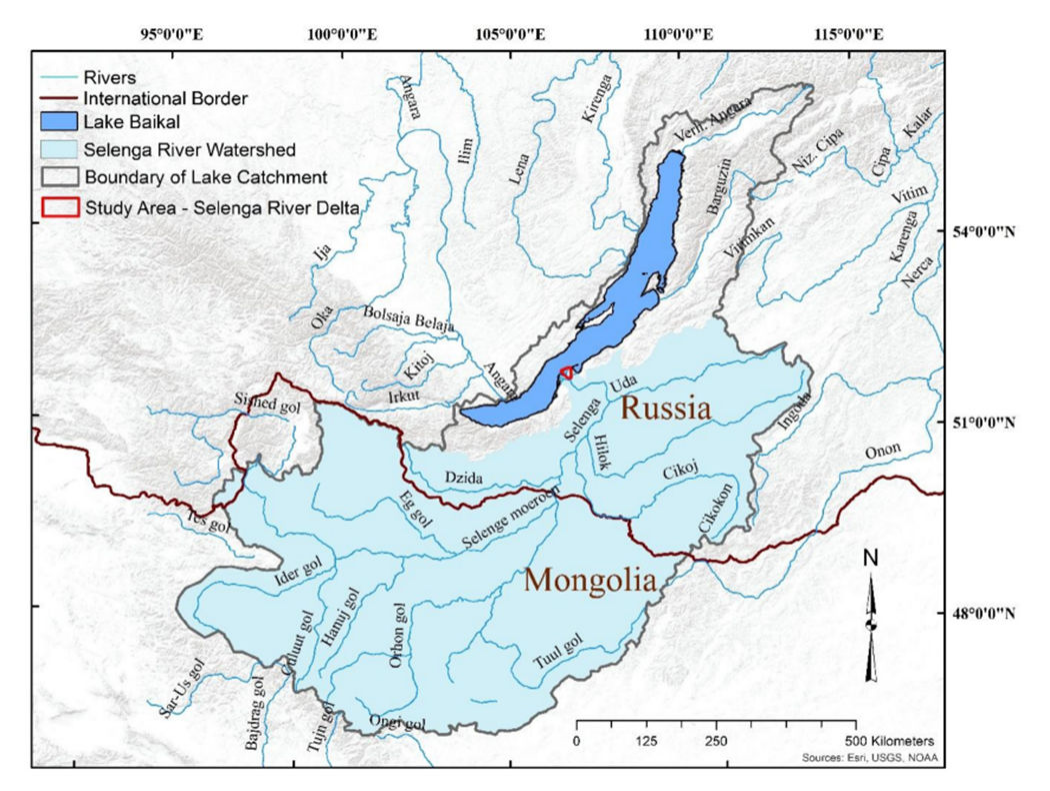
The watershed contributing to the study area, the Selenga River Delta into Lake Baikal, Russia.

The study area includes the Kabansky Nature Reserve and the surrounding area within the ~600 km^2^ Selenga River Delta in southeastern Siberia, Russia [59]. The delta has a variety of wetland habitats, from open water to emergent marshes, shrub scrub, forested wetlands, and mixed habitats [60,61]. The delta traps suspended sediments and filters excess nutrients, heavy metals, and other pollutants from the Selenga River [59,62]. The Selenga River Delta was defined as a Ramsar Wetland of International Importance in 1994 for its significant flora and fauna [63]. The Selenga River, which provides sediment and water resources to maintain the delta, is the largest tributary to Lake Baikal and comprises ~82% of the lake’s watershed [64] (Figure 1). The Selenga River supplies approximately 50% of water runoff and 60% of sediments to Lake Baikal [65]. Lake Baikal, the deepest and most voluminous freshwater lake in the world, holds approximately 20% of all liquid freshwater on Earth [63]. Located at a relatively high latitude in a semi-arid environment, the Selenga River Delta is particularly sensitive and vulnerable to climate change and water abstraction [66,67].

### 2.2. Spatial Data, Preprocessing, and Initial Field Classifications

Two cloud-free Ortho-Ready Standard (OR2A) WorldView-2 (WV2) images from 25 June 2011, and 3 July 2011, were acquired for this study. There is a 5-km-wide overlap area between the 2 images. The WV2 multispectral imagery has 8 spectral bands (2.0 m pixel size): coastal (400–450 nm), blue (450–510 nm), green (510–580 nm), yellow (585–625 nm), red (630–690 nm), red-edge (705–745 nm), near infrared-1 (NIR1, 770–895 nm), and near infrared-2 (NIR2,860–1040 nm), as well as a panchromatic band (0.5 m pixel size).

**Figure 2.**
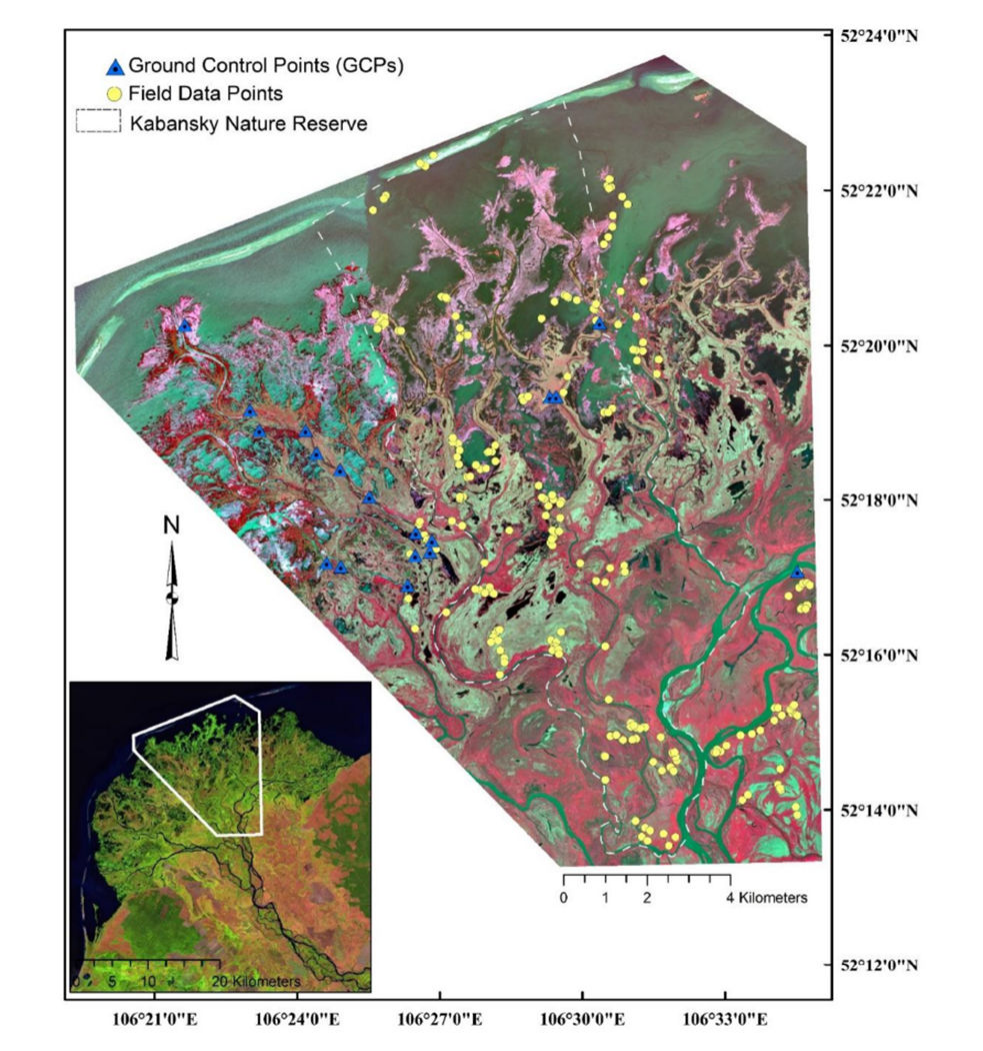
WorldView-2 false-color composite (near infrared-1 (NIR1), red, green) of the study area showing the spatial location of the field-sampling locations and ground control points. Inset image shows the Selenga River Delta in Lake Baikal and the study area boundary.

During the 2011 and 2012 field expeditions (described below), we collected 21 ground control points (GCPs) corresponding to relatively permanent features easily identified on the WV2 images, such as building corners, single isolated trees, “corners” (i.e., high curvature points) of river channels, and tree stands. GCP location data were collected using the Trimble Nomad and Yuma GPS receivers (Trimble Navigation Limited, Westminster, CO, USA) with 100 points averaged for each location. The locational geo-accuracy error of the WV2 images was less than 5 m as confirmed by the 21 GCPs. To make the 2 images radiometrically comparable, the digital number values of the original WV2 images were calibrated and converted to the top-of-the-atmosphere reflectance values, which accounts for solar geometry differences at the 2 image acquisition dates. The combination of the 2 images covers an area of 215 km^2^, including the entire Kabansky Nature Reserve and its surrounding area (Figure 2). The WV2 data were not ortho-rectified.

We conducted a preliminary ISODATA classification to inform our field study in 2011, initially selecting 24 classes with sufficiently high Jeffries-Matusita (J-M) separability values [68]. After the field expedition in 2011, we conducted a maximum likelihood classification to inform the 2012 field expedition, merging some classes to create a 22-class wetland landscape, as detailed in Lane et al. [60,61].

### 2.3. Field Data

A total of 228 field data points were collected over the 2011 and 2012 expeditions (see Figure 2). The field data were collected in July 2011 and July 2012. Homogeneous polygons derived from the preliminary classifications (described in Section 2.2) were visited by teams of botanists and ecologists. At least 3 unique polygons for each class were sampled across the study area, accessed by boat, hiking, and/or wading. Vegetation and structural data were collected at each point within a 100 m^2^ sampling frame established for each sampling point. Information collected included identification of the species and relative abundance of all plants with ≥10% coverage and ancillary information, including water depth, nonvegetation abundance (i.e., bare ground), and substrate composition within the sampling frame. Photographs were taken from the center of each polygon in the 4 cardinal directions. No major changes occurred in the wetland systems between the 2 field data collection periods. For this analysis, species-level abundance data were collapsed to the genus level to facilitate comparisons among the classifiers.

### 2.4. Regions of Interest

Subsequent to the field expeditions, regions of interest (ROIs) were established using ENVI (Harris Geospatial Solutions, Herndon, VA, USA, version 5.3) around each field point (i.e., the center of each polygon) for use in the classification. Each ROI was approximately 28 pixels in size to approximate the field sampling frame, and was normally centered on the field point. Occasionally, an ROI was moved slightly off the field point to account for spectral noise and/or speckling. The pixels of each ROI were ascribed that ROI’s value for the wetland class (i.e., one of 22 classes based on the 2012 field analyses) and assigned the spectral and ancillary data of the pixel values described in Section 2.5. We then randomly sampled ROIs for training (*n* = 158) and testing (*n* = 70) datasets. Each wetland class had, on average, 7 ROIs for training and 3 for testing (training range: 2–12 ROIs; testing range: 1–5 ROIs). The partition of training and testing datasets was performed by visually inspecting the point distribution so that the training and/or testing datasets were distributed in space to maximize the distance between the points, thereby minimizing the chances of spatial autocorrelation. A total of 6262 pixels for training and 2773 pixels for testing were identified.

### 2.5. Creating Spectral Metrics

Various spectral and landscape metrics can increase our ability to accurately discretize the landscape [69,70]. We therefore calculated additional characteristics to parametrize our models. Univariate Pearson’s product-moment linear correlation analyses among the variables were also conducted (Table 1). Three spectral metrics expected to improve classification accuracy were calculated based on differing ratios between spectral bands: the Normalized Difference Vegetation Index (NDVI, [71]), Normalized Difference Water Index (NDWI, [72]), and Normalized Difference Soil Index (NDSI, [73]).

The NDVI is a well-established indicator for the presence and condition (i.e., abundance, vigor, and health) of vegetation [71]. The radiometrically calibrated reflectances of WV2 band 5 (Red) and band 7 (NIR1) were used to compute the NDVI. Healthy and abundant vegetation reflects strongly in the near-infrared portion of the spectrum while absorbing strongly in the visible red light portion, yielding high positive NDVI values. Sparse, stressed, and flooded vegetation has smaller positive NDVI values. Open water bodies yield negative values due to larger red reflectance than NIR. The NDVI values for bare soil ground are near zero due to their similar reflectance in both bands. The NDVI value in the study area ranges from −0.46 to 0.87 (data not shown).

We also calculated the NDWI following Wolf [73], based on the reflectance of WV2 band 1 (B1, Coastal) and band 8 (B8, NIR2) as ((B1 − B8)/(B1 + B8)). Water features in the NDWI will typically have positive values, while soil and vegetation will have negative or zero values [74]. NDWI values in the study area range from −0.61 to 0.83 (data not shown).

The NDSI discriminates soils from other background objects and was computed using WV2 shortwave bands 3 (B3, green) and 4 (B4, yellow; [73]) as ((B3 − B4)/(B3 + B4)). It is used to quantify dissimilarities of vegetation cover density and soil properties [75]. Despite the need for the shortwave infrared (SWIR) band to generate an index capable of discriminating soils from other objects (i.e., brightness intensity of soils is higher at longer wavelengths), the ratio of the aforementioned two visible bands of the WV2 dataset was consistently found to be a robust index in similar applications capable of discriminating soil from other image objects [73,76]. Exposed soils, non-photosynthesizing vegetation, and inundated areas tend to exhibit negative and low positive NDSI values, while healthy vegetated areas have higher positive NDSI values. For the study area, the NDSI ranges from −0.41 to 0.54 (data not shown).

We also calculated a texture metric [77] to characterize the spatial structure of the wetland vegetation and habitat [78]. We quantified the homogeneity texture variable using Gray Level Co-occurrence Matrices [79] with a grayscale quantification level of 64 and a 3 × 3 processing window. Water surfaces and emergent grasses have relatively smooth texture and subsequently high homogeneity values. Forested and scrub-shrub habitats have relatively rough texture and low homogeneity values, providing a contrast in habitat classification. Textural values in the study area range from 0.0 to 1.0 (data not shown).

### 2.6. Landscape Metrics and Topographic Data

To represent spatial associations and domain knowledge [69,70], we also introduced three hydrogeomorphic metrics: landscape/topographic position, distributary stream channels, and surface depressions. We further assigned elevation data to each ROI.

#### 2.6.1. Landscape/Topographic Position Variable

The Selenga River Delta has a protruding fan shape with a loam- and sand-rich soil. The outer boundary of the delta is configured by a chain of long sand spits (depositional sandbars) (see Figure 2). Between the sandbar chain and the vegetated delta front is a subaqueous deltaic plain composed of silt and clay sediments. Hydrogeomorphologically, the delta is composed of low, central, and high portions. The low, northern, peripheral portion of the delta is subjected to regular floods [80]. The central floodplain and southern high islands are only flooded during high floods, and the floodplain terraces are typically not affected by floodwater. The floodplain terraces have flat or slightly undulating surfaces, which is complicated by the natural levees along former and present river channels [80].

We used the depositional sandbars described above to delineate the peripheral “shoreline” extent for the Selenga River Delta. Then the shortest distance from each WV2 pixel to the delta shoreline was created as an ancillary geographic information system (GIS) layer to indicate the landscape/topographic position along the relevant gradient in the broader landscape from lowlands, to midlands or central areas, to highlands. This variable is also closely related to the magnitude of seiches created from strong prevailing winds across the lake.

#### 2.6.2. Distance to Stream Channels

The Selenga River enters Lake Baikal via the Selenga Delta wetland through a complex array of channels that are active and ice-free for approximately six months of the year. The inter-distributary bays are filled with sandy deposits frequently redistributed by waves. The delta consists of sandy lobe islands separated by numerous elongated and bifurcated channels [81]. Numerous distributary channels with natural levees separate extensive marshes, and lakes, channel cutoffs, and oxbows are abundant on the lobate islands. Natural levees with loamy sandy and sandy alluvial deposits and meadow marshes are widespread within the lower portion of the delta.

We extracted the stream channel features from the multispectral WV2 imagery sharpened by its panchromatic band using the NDWI thresholding method. The shortest distance from each WV2 pixel to the stream channels was created as another ancillary GIS data layer. The distance to distributary channels was used as a hydrogeomorphic variable to indicate the spatial relationship (proximity) to stream levees, closely related to elevation and flooding frequency, which has a controlling effect on wetland vegetation.

#### 2.6.3. Distance to Depressional Features

Ponds, lakes, and marshes were extracted from the pan-sharpened WV2 imagery to indicate proximity to hydrogeomorphic features characterized as surface depressions. Depressions (or the ponds, lakes, and marshes that form in areas relatively distal to the stream network) are formed through ice-block and water-scouring actions, the magnitude of which is controlled by the energy of the water. We hypothesized that distance to a surface depression would be a hydrogeomorphic proxy for a combination of exposure to water-energy and sediment transport and could inform wetland vegetation typology. Similar to the stream channel extraction, we extracted the depressional features (e.g., lakes, ponds) using the NDWI thresholding method along with shape indices (see, e.g., [82]). The shortest distance from each WV2 pixel to the depressional features was created as another ancillary GIS data layer.

#### 2.6.4. Surface Elevation

A digital surface-elevation dataset from the Advanced Spaceborne Thermal Emission and Reflection Radiometer-Global Digital Elevation Model (ASTER-GDEM, 30 m spatial resolution) was used as an auxiliary predictor variable. As with the aforementioned landscape metrics (predictors), elevation was expected to be a proxy for wetness, with areas of lower elevation being prone to longer hydroperiods. The dataset was projected (WGS84, UTM Zone 48 Northern Hemisphere) and resampled to a WV2 native pixel resolution of 2 m. The elevation of the study area ranged from 419 to 484 m above sea level, with an average elevation of 446 m.

### 2.7. Decision-Tree, Rule-Based, and Random Forest Classification and Assessment

#### 2.7.1. Overview

We contrasted three different classification approaches: DT, RB, and RF. The classification models were constructed iteratively, adding information content at each step. Each iteration was considered a “test,” and the overall accuracy of that iteration and approach contrasted between the three methods. Models were initially constructed using the four traditional bands: Test 1: B2 (Blue), B3 (Green), B5 (Red), B7 (NIR1). We then added the WV2 coastal band (B1) as Test 2, as Lane et al. [60] determined that this band could facilitate open-water and vegetated habitat discrimination in wetlands. The full eight bands available in WV2 were analyzed (Test 3), and we then iteratively augmented the eight-band stack in a stepwise approach with the derived indices:B1–8 plus Test 4: NDVI, Test 5: NDWI, Test 6: NDSI, Test 7: texture, and Test 8: elevation. We tested WV2 B1-B8 plus all spectral metrics (e.g., NDVI, NDWI, etc.) initially without boosting (Test 9), and then adding the “boost” function described below (Test 10) for the DT and RB approaches only. We analyzed B1–B8 plus the four spectral metrics and all three hydrogeomorphic variables (Test 11); we subsequently analyzed the same 15-layer stack using the “boost” function for the DT and RB approaches (Test 12). We analyzed B1–B8 plus the four spectral metrics and all three hydrogeomorphic variables (Test 11). We subsequently analyzed the same 15-layer stack using the “boost” function for the DT and RB approaches (Test 12). We analyzed B1–B8 plus four spectral metrics, three hydrogeomorphic variables, and elevation initially without boosting (Test 13), and with boosting for the DT and RB approaches only (Test 14). Lastly, since we intended to develop classification models with the minimum number of input variables possible (thereby decreasing data dimensionality) while achieving the highest possible overall accuracy, we systematically worked through model parameterization to develop the most parsimonious models by removing highly correlated variables (see Table 1; Tests 15 and 16).

#### 2.7.2. Decision-Tree Classification

Both DT and RB models were developed using C5.0 [83]. DT classification employs a hierarchy of rules in an automated, top-down, dichotomous fashion [84–86]. C5.0 uses a recursive partition procedure to build a binary DT. The DT is composed of different levels of nodes: a root node, a set of internal nodes (branches), and a set of terminal nodes (leaves). The root node or whole dataset is divided (split) into more homogeneous groups. The split at each internal node of a tree is defined by a single predictor variable based on statistical analysis of the training data [43]. The split is made based on determining predictor variable values with the most discriminatory power, measured by the information gain ratio with iterative predictor-variable values in C5.0 [36]. Split nodes subsequently contain only part of the data and can further be divided until an end node (leaf) is reached, where no further split is possible or desired. Both DT and RB models can be improved through boosting, wherein misclassified leaves in the final model are reanalyzed and the model reiteratively runs in an attempt to properly classify these errors.

**Figure 3.**
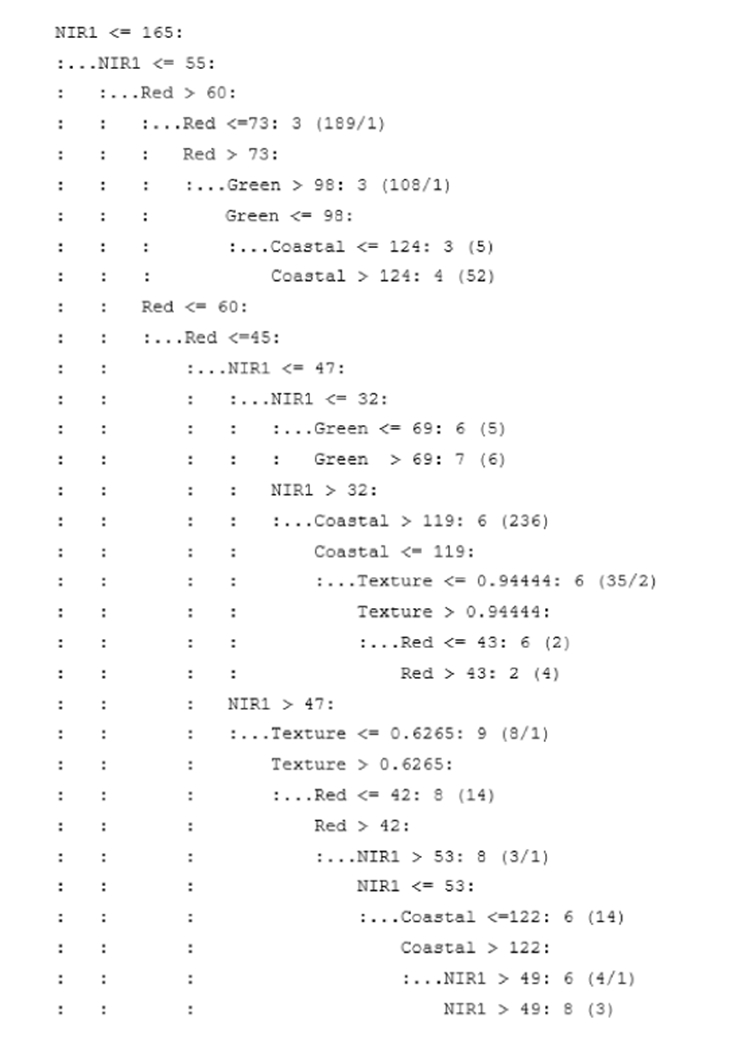
An example of the decision-tree outcome for classifying wetlands of the study area.

The DT was constructed in two steps. First, a large tree was grown to fit the training data closely. Then, the tree was pruned (or winnowed) to remove attributes that affected the error rate. This pruning process attempted to correct overfitting errors and reduce the tree size [36,83,87]. DT and RB models were boosted in Tests 10, 12, 14, and 16, wherein the misclassified leaves in the final DT were reanalyzed and the model was reiteratively run in an attempt to properly classify these errors. Boosting was conducted for 10 trials or until the model performance failed to improve as measured by the error rate. The portion of the DT from the 5-layer stack (WV2 coastal, green, red, and NIR1 bands plus texture) is displayed as a branching dichotomous tree in Figure 3.

#### 2.7.3. Rule-Based Classification

**Figure 4.**
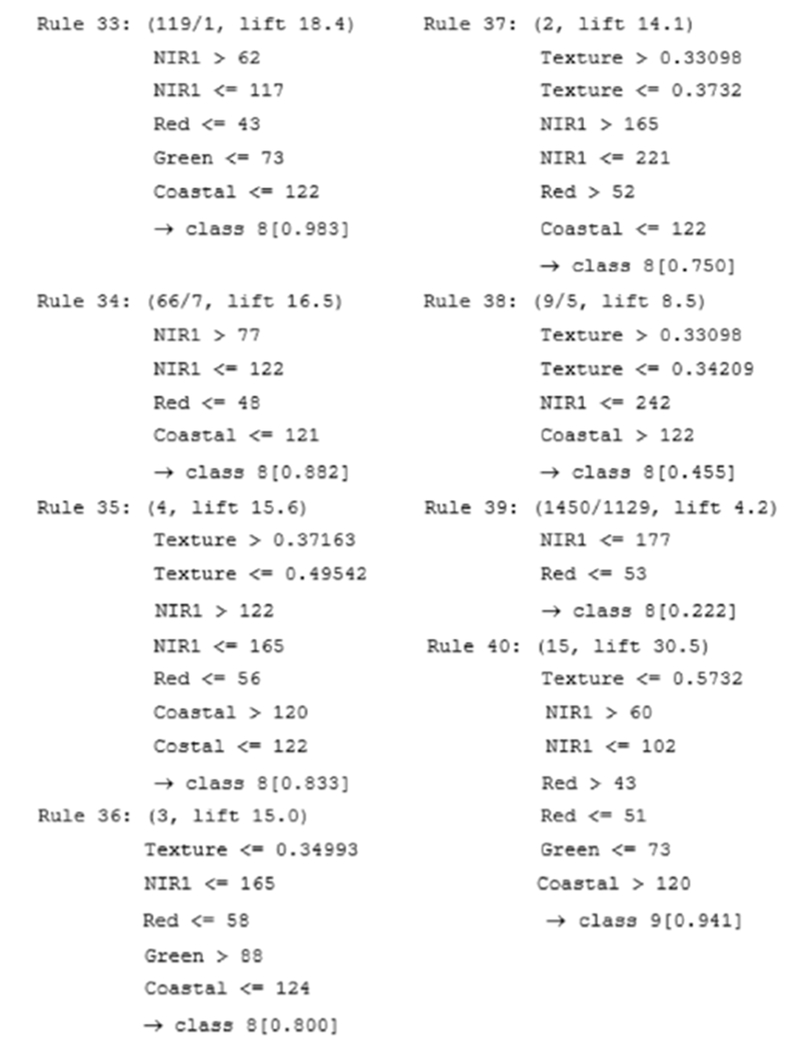
An example of the rule-based approach for classifying the Selenga River Delta wetlands.

The DT developed in 2.7.2 (including the boosting conducted in Tests 10, 12, 14, and 16) was transformed into a simpler set of “if-then” rules in C5.0 by creating “rulesets” in the algorithm. The ruleset generated from the DT has fewer rules than the number of leaves in the decision tree, thus it is a more compact and simpler representation [88]. Since each conditional logic rule describes a specific context associated with a class, it is relatively easy to examine, validate, and interpret the ruleset. The “if-then” logic rules make the connection between wetland classes and their predictor variables. A portion of the ruleset when using the same five-layer stack as in Figure 3 is shown in Figure 4.

#### 2.7.4. Random Forest Classification

In contrast with the single optimal tree built using the entire training dataset and all of the predictor variables in the DT and RB approaches, RF creates an ensemble of trees that each provides a “vote” to select the best classification approach. That is, class membership in a DT is decided by a single tree, whereas the majority of votes from the assemblages of trees built by RF decide the class assignment of a given pixel. We used the randomForest package [51] in the R statistical software environment (RStudio, Inc., Boston, MA, USA, version 1.0.143). Each RF tree was built by training each DT (ntree) with a random subset of the predictor-variables (mtry) from the training dataset with a replacement [49,89]. Based on the preliminary analyses, we selected RF models comprising ntree = 1000 trees, 1000 bootstrap (or “out-of-box”, OOB) samples to assess internal model error, and tested multiple predictor variables (i.e., mtry = the square root of the total number of input variables, either 2, 3, or 4) at each split as we tested from the simplest to the most complex model (i.e., 4-, 9-, and 16-layer stacks).

#### 2.7.5. Accuracy Assessment

Each ROI pixel in the training dataset (i.e., 158 ROIs composed of 6262 pixels) was used to construct DT, RB, and RF models, ranging from the relatively simple (i.e., four bands as input) to complex (i.e., 16 data layers including eight bands, four spectral metrics, three hydrogeomorphic metrics, and elevation). The holdout 70 ROI (2773 pixels) as a validation dataset was used to assess the prediction accuracy of the approach across all 16 tests, resulting in an average of 103 validation pixels generated per class for each of the 22 classes in the evaluation. Performance measures included overall accuracy, class-wise producer’s accuracy (PA; errors of omission), and class-wise user’s accuracy (UA; errors of commission). We quantitatively assessed if the observed difference in the classification accuracies between the “best” or most accurate application of each of the approaches was statistically meaningful using 95% confidence intervals [90].

## 3. Results

### 3.1. Field Data Collection

Fifty-two different plant genera were found at ≥10% coverage in our 228 sampling frames. Members of the genera *Equisetum* and *Carex* were most commonly found, with 58 and 45 sites, respectively. *Nymphoides* (41 sites) and *Salix* (27 sites) were also commonly encountered. Open water (100 sites) and thatch (45 sites) were also noted to cover ≥10% of the sampling frame in the field sites. Fourteen genera were encountered only a single time. Because the main goal of this paper is to determine the effectiveness of the DT, RB, and RF approaches, we do not further describe the ecology of the wetland classes here (but see [60,61]).

### 3.2. Decision-Tree, Rule-Based, and Random Forest Classification Accuracy and Complexity

We examined the performance of the DT, RB, and RF approaches based on a random sample of 2773 validation pixels, determined from field-sampled sites and independent of the training pixels. Performance measures included OA, class-wise PA (errors of omission), and class-wise UA (errors of commission).

#### 3.2.1. Classification Accuracy

The training and testing dataset ROIs were independent and widely distributed across the study area, minimizing the potential for spatial autocorrelation by predictor variables. As shown in Table 2, the OA on the testing dataset ranged from 54.8 (Test 11: 15-layer stack and RB classification) to 81.2% (Test 16: 5-layer stack and RF classification). The highest OAs for DT and RB were 80.7% (Test 16) and 80.0% (Test 10), respectively. Both tests achieved the highest accuracy with boosted classification. Test 16 was also the highest-performing RF classification (81.2% OA). We assessed the classification accuracy of the best-performing models for each approach, and, as the 95% confidence intervals overlapped, we found no significant differences between the test results (DT Test 16, RB Test 10, and RF Test 16).

#### 3.2.2. The Effects of Additional Bands and Input Parameters

Similar to Lane et al. [60], we found that the addition of available spectral bands in WV2 increased OA (Tests 1 and 3) across the DT, RB, and RF models by 6.2%, 8.2%, and 3.6%, respectively. The improvements from adding derived indices (i.e., NDVI, NDWI, NDSI) vacillated across the three approaches, with no marked increase in overall accuracy (e.g., Tests 4 to 6), but adding texture increased OA by 3–5%, depending on the approach (contrasting Test 3 with Test 7). As the 22 wetland classes were composed of different hydroperiods and inundation regimes, vegetative structures, and soil characteristics that affect the spectral signal received by the WV2 sensors, particular classes (e.g., those with abundant forest structure) might respond with substantial increases (or decreases) in comparative accuracy between different tests.

However, contrary to our expectations, including hydrogeomorphic variables and/or elevation data resulted in a marked decrease in OA across all approaches (e.g., contrast Test 3, Test 8, and Test 11 with Test 14 across all approaches in Table 2). For example, including the elevation dataset decreased the OA by 11.5%, 12.8%, and 1.4% for DT, RB, and RF, respectively (e.g., contrast Test 3 and Test 8 in Table 2). Similarly, including three hydrogeomorphic variables (landscape position, distance to stream channels, and distance to depressional features; Sections 2.6.1–2.6.3) along with the eight multispectral bands and four spectral indices resulted in a decrease in OA by 21.9% for DT and 23.9% for RB (Test 9 and Test 11, Table 2). With the boost function, there were similar decreases in OA, 19.9% for DT and 21.1% for RB (Test 10 and Test 12). A similar decrease was also observed for RF, though by a smaller amount, 5.9% (see Table 2).

Removing highly correlated (|r| ≥ 0.89) predictor variables (e.g., B2, blue; B4, yellow; B6, red-edge; and B8, NIR2; see Table 1) from Test 7 yielded approximately the same OA results as shown in Test 16 (see Table 2): 80.7% for DT and 81.2% for RF. Consequently, for DT and RF, the models that combined parsimony and accuracy were built using a five-layer stack of input variables (Test 16: B1, coastal; B3, green; B5, red; and B7, NIR1; and texture). Using the same parsimonious predictor variables, a lower OA of 77.8% was achieved for RB (i.e., in contrast with Test 10, 80.0% RB OA).

## 4. Discussion

### 4.1. Random Forest as the Classifier of Choice

In all iterations (see Table 2), the RF model outperformed both DT and RB. In addition, the RF model appeared to better handle an increasing number of predictor variables that resulted in higher OA, demonstrating its ability to effectively process complex and highly dimensional datasets. Furthermore, RF provides useful information to the end user in terms of mean decrease in Gini (MDG, [49]), a measure of the relative importance of different predictor variables affecting overall accuracy. Though not the focus of this study, the MDG indicates that NIR1 (B7) has the greatest effect on overall model accuracy in the best RF model (Test 16, data not shown). Therefore, end users wishing to increase OA could consider ensuring that NIR1 (B7) is used in models addressing their study area, focusing the development of additional indices to improve OA on other information content in the spectral data (e.g., focusing on the effects of soil reflectance, or calculating texture metrics).

DT and RB models were able to approximate the RF results (e.g., OA between all three approaches was within 0.5% in Test 10 and 3.5% in Test 16), and overlapping confidence intervals indicated no significant differences between the different approaches [90]. DT and RB were only able to achieve near parity with RF through the use of the boost function in C5.0. Similar to the voting aspect of RF, the boost function predicts a given class assignment by using the majority of votes from multiple classifiers as opposed to a single tree or ruleset. Moreover, similar to the random selection of input variables (mtry) by RF, the ability to make subsets of the predictor variables to construct the DTs and rulesets was achieved through the “winnowing” mechanism of the C5.0 package. However, relative to RF, these different steps in the DT and RB approaches require additional processing and user input.

Throughout the literature we found many instances of users classifying landscapes with DT and RB methods, but we found only one, Rodriguez-Galiano et al. [91], that contrasted the outcome between a DT and RF. In their study, they mapped 14 land-cover categories using Landsat TM and ancillary datasets with OA of 86% for the DT approach. Similar to our efforts, RF increased OA to 92%, and RF also outperformed DT/RB when model parsimony was optimized.

However, RF is considered a “black box” model, wherein much of the algorithm is performed in the virtual background [92]. That requires accepting the outcomes or laborious efforts to unpack the algorithm. In addition, RF OA can be sensitive to the distribution of ROIs; unequal distribution can affect the RF outcome relative to a balanced approach [93,94]. However, it appears the benefits of RF outweigh the detriments, and as such we suggest that end users strongly consider using RF in their classification applications.

DT and RB may be useful when the rules and/or tree nodes and splits are contextually relevant and useful to end users [84], versus the aforementioned “black box” nature of RF resulting in difficult rule extraction and model comprehension. Should end users rely on DT and RB approaches, we recommend using the boost and winnowing functions of the C5.0 package, which could improve the OA of the DT and RB classifications.

### 4.2. Overall Accuracy with a Large Suite of Classes

Whereas land cover classification is common, using remotely sensed data to specifically assess and conduct wetland classification is somewhat rarer (see, e.g., [95] for a detailed review of approaches). Furthermore, we have found that most wetland classifications limit the classes to a relatively small number, depending on the end goals of the user (e.g., 10 to 11 classes, [9,96]). The deltaic wetland we studied was discriminated into 22 classes, which set a high bar for achieving acceptable overall classification accuracy. We would have expected higher OA if we had fewer classes, or if we targeted certain classes and perhaps merged them together.

The 22 classes had high Jeffries-Matusita (J-M) separability values [68] Table 3). These values range from 0.00 to 2.00, with values <1.0 suggesting poor separability and values approaching 2.0 indicating high separability; we found 18 contrasts with J-M values less than an arbitrary 1.75, and only two instances where the J-M values were <1.20 (Class 8 and Class 9; Class 21 and Class 22). As evidenced by the above findings, lower J-M values typically occurred along neighboring classes, as might be expected. Dubeauet et al. [97] classified a headwater wetland ecosystem in the Dabus River basin, a large tributary of the Abay-Blue-Nile River in Ethiopia, using Landsat TM and an RF approach. Similar to our findings, they found that among the eight wetland types and three upland classes, the greatest confusion was between similar neighboring plant types and vegetation structures (e.g., greater confusion within herbaceous classes than between herbaceous and woody/shrub or open water classes). In general, our table-wide J-M average was 1.95, suggesting that the 22 classes were well discriminated by the high-resolution WV2 data.

### 4.3. Metrics, Classes, Spectral Bands, and Hydrogeomorphic Variables

Using vegetative, soil, and hydrologic indicators based on various band combinations (NDVI, NDSI, and NDWI, respectively) did not markedly improve the classification. This is similar to the findings of Berhane et al. [47], who reported decreased classification OA (and/or no meaningful change) when exploring the influence of over 30 predictor variables on classification accuracy (using Quickbird imagery), including the NDWI, NDVI, and a functionally similar soil metric, the NDSI. However, they did find increased OA when incorporating a metric functionally similar to the NDWI, the Water Ratio Index [98]. Thus, though the primary goal in this study was to contrast the three classification approaches, in order to fully and accurately characterize the wetland landscape, full consideration of a multitude of band combinations should be explored (see, e.g., [47], Table 1, for a list of potential metrics to consider; see also [95]).

The results of this study, as well as those of Franklin and Peddle [78], Lane et al. [60,61], Berhane et al. [47], and others, show that including the homogeneity texture variable, calculated using the Gray Level Co-occurrence Matrices [79], did substantially improve classification OA. Water surfaces and emergent grasses have relatively smooth texture and subsequently high homogeneity values. Increasing vegetative structure, such as that found in forested and shrub-scrub wetland habitats, imparts relatively rough texture and low homogeneity values, apparently providing a useful contrast in wetland vegetation and habitat classification. These structural features of different wetland vegetation types, as determined by the texture measure included in this study, are thus recommended to provide a useful and additive metric to improve classification outcomes.

**Figure 5.**
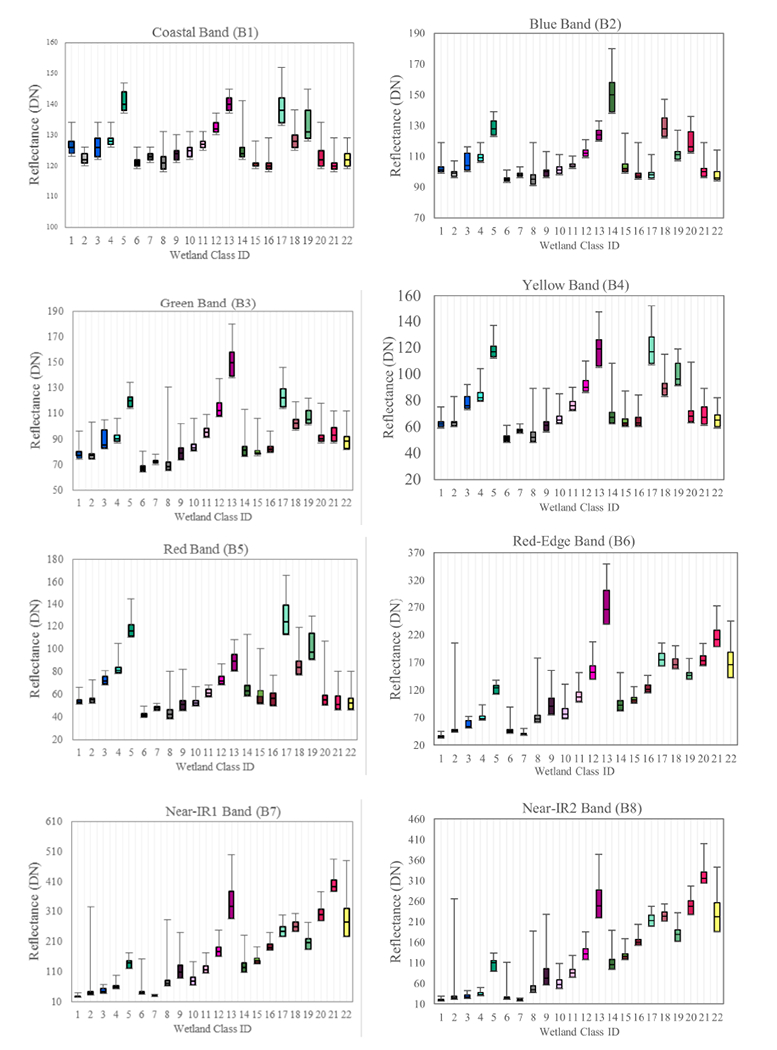
Median WV2 band distribution indicates strong discriminatory power between classes.

**Figure 6.**
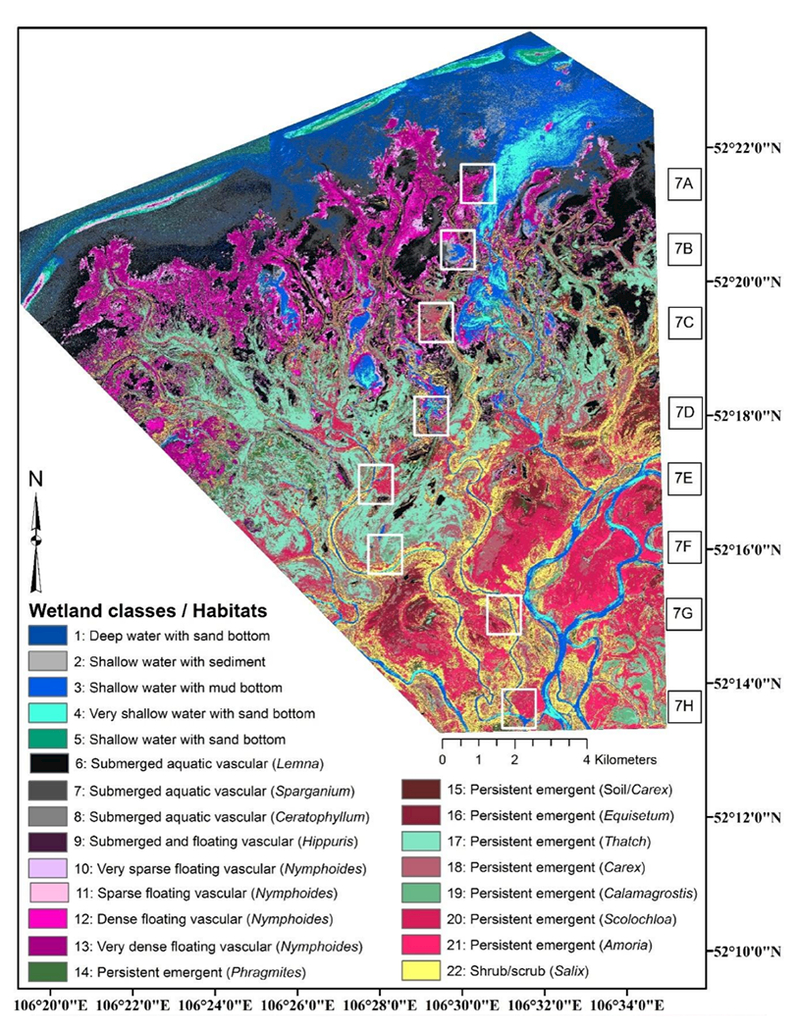
The RF-classified study area. Classes in the legend were attributed based on wetland plant abundance, water depth, and substrate composition (see, e.g., [60]). The north-to-south, wetter-to-drier boxes in Figure 6 are further discussed in Figure 7A-H.

**Figure 7.**
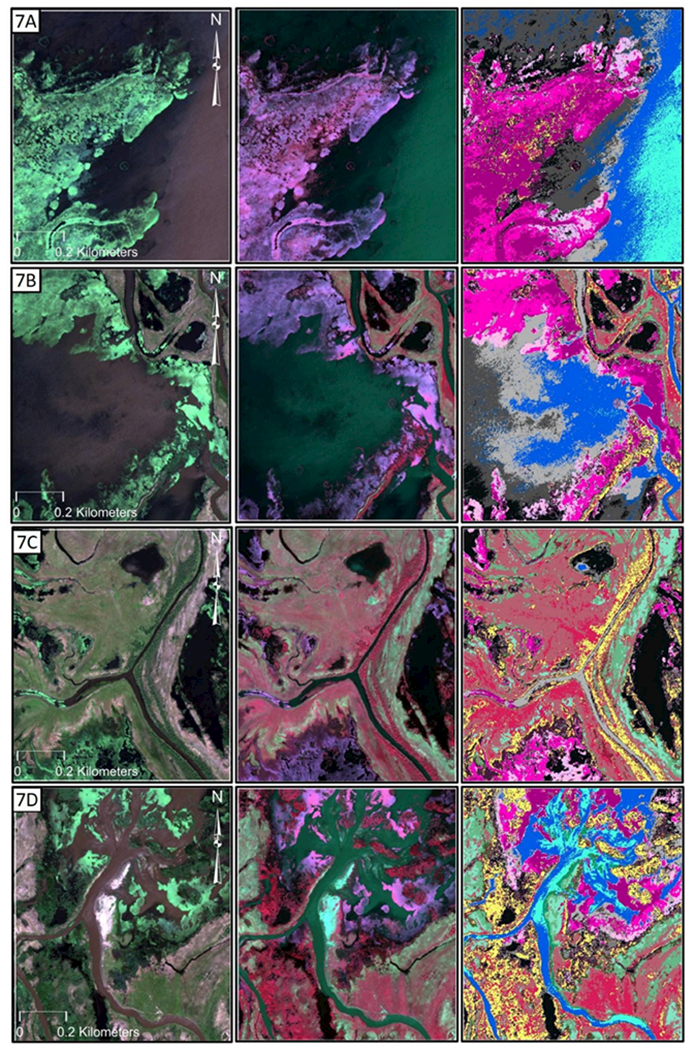

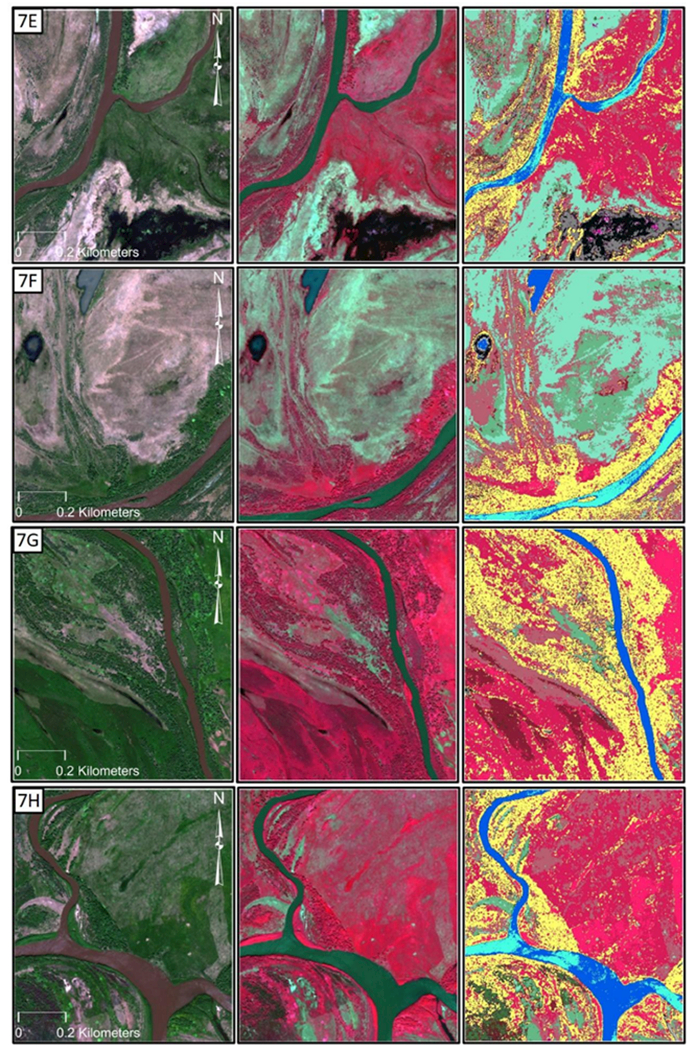
**(A-H).** The vegetation of the Selenga River Delta follows a north-to-south and wetter-to-drier gradient, as evidenced by the abundance of different wetland classes within the white rectangles in Figure 6. The images are combined WV2 bands 532 (**left**), bands 753 (**middle**), and the wetland classification thematic map (**right**) using the legend in Figure 6.

There appears to be relatively strong discriminatory power among the spectral bands when used in combination; exploring the median distribution of the WV2 bands across the 22 classes (Figure 5) supports this statement. In other words, the fact that the derived metrics, notwithstanding texture, did not markedly improve OA appears to be a manageable situation, wherein these data alone can provide useful information to the end user. This is further supported by the relatively high ~75% OA for the eight WV2 bands when analyzed as an 8-band stack (Test 3, Table 2). For instance, NIR1 and NIR2 have values closer to zero for Classes 1–7 (excluding Class 4) and increase linearly through to Class 22, with relatively minor overlap between median reflectance values among the wetland classes in this study. Interestingly, the remaining six bands indicate that the classes may be visually discriminated into approximately four clusters: Classes 1–5, Classes 6–13, Classes 14–16, and Classes 17–22; these may be further explored to better understand wetland classification and ecology. For instance, we found that our classification followed a wet-to-dry gradient, as evidenced by the vegetation data in Figure 6 and more closely explored in the wet-to-dry, north-to-south gradient evidenced in Figure 7A–H. Deeper waters and submerged vegetation manifested in the northern portion of the study area, and emergent vegetation and facultative upland genera were found in the southern portions.

With this distribution of wetland classes following a wet-to-dry gradient, we were surprised that our hydrogeomorphic and elevation data did not improve the classification. Indeed, including these variables decreased overall accuracy (e.g., Test 9 versus Test 11, Table 2; see also Test 3 and Test 8 for elevation effects). The elevation effect may be ascribed to the relatively flat nature of the delta, where the granularity of the topographic terrain provided no meaningful or discernable attribute information about the wetland classes to the classifiers, but rather imparted noise. We had expected elevation to play an important role, as even slight differences in elevation can dramatically affect inundation patterning, soil biogeochemistry, and vegetative structure. Perhaps if a higher-resolution DEM was found for the study area, we might find it a useful input variable.

Similarly, including three hydrogeomorphic variables (landscape position, distance to stream channels, and distance to depressional features; Sections 2.6.1–2.6.3) decreased overall accuracy by approximately 20% for DT and RB and 5% for RF (e.g., Tests 9 and 11, and Tests 10 and12 in Table 2). The complexity of the delta, wherein stream networks and channels are constantly migrating, affecting inundation patterning and water clarity and modifying hydrologic gradients, likely also meant that the granularity of our hydrogeomorphic variables was too coarse. We suspect that further refinement of hydrogeomorphic variables may improve their influence on OA (e.g., incorporating stream width, flow direction, and proportion of flow, as an energy surrogate, may correlate with the distribution of the wetland habitats).

## 5. Conclusions

In this paper, we systematically and comprehensively evaluated the utility of three nonparametric machine-learning algorithms (DT, RB, and RF) for effective supervised classification of 22 complex freshwater deltaic wetland vegetation and aquatic habitats in the Selenga River Delta of Lake Baikal, Russia. The use of WV2 multispectral bands, derived spectral indices, and ancillary data was optimized through iterative modeling and predictor variable selection to achieve a satisfyingly accurate working model. Our analysis shows that DT, RB, and RF classification methods provide a suitable framework to combine different types of data sources, accommodating image-derived indices and ancillary hydrogeomorphic variables in addition to image spectral bands and elevation datasets. The OA of the DT, RB, and RF classification methods ranged from 54.8 to 81.2%. The RF classification outperformed both the DT and RB classifications; performance is approximately equal when boost and winnowing functions, available in the C5.0 package, were used. We conclude that RF can be used as the classifier of choice in most cases, except, potentially, in situations where end users require narrative rules to best manage their resources. That would call for the DT or RB approach, though the breadth and abundance of rules (upwards of 140 rules or tree leaves to achieve OA ≥80%; see Table 2) may be daunting. Including a texture metric (homogeneity) substantially improved the classification OA. However, we were surprised that including vegetation/soil/water metrics (based on band combinations), hydrogeomorphic, and elevation data layers did not markedly improve OA. This may be a result of the complexity of the deltaic wetland system, which requires finer-resolution spatial data to meaningfully improve classification models.

## Figures and Tables

**Table 1.** Correlation matrix of predictor variables. Linear correlations (|r| ≥ 0.89) are shaded gray.

Predictor	Coastal	Blue	Green (B3)	Yellow	Red	Red-Edge	NIR1	NIR2	NDVI	NDSI	NDWI	LP	SD	DTS	Texture
	
	(B1)	(B2)		(B4)	(B5)	(B6)	(B7)	(B8)							
Blue (B2)	0.96														
Green (B3)	0.87	0.88													
Yellow (B4)	0.93	0.96	0.93												
Red (B5)	0.87	0.95	0.79	0.94											
Red Edge (B6)	0.42	0.46	0.75	0.57	0.40										
NIR1 (B7)	0.21	0.27	0.56	0.38	0.26	0.95									
NIR2 (B8)	0.21	0.29	0.55	0.39	0.29	0.93	0.99								
NDVI	−0.06	−0.02	0.29	0.09	−0.03	0.79	0.86	0.86							
NDSI	−0.65	−0.69	−0.41	− 0.70	−0.80	0.02	0.15	0.10	0.36						
NDWI	−0.21	−0.28	−0.52	−0.39	−0.30	−0.89	−0.94	−0.96	−0.92	−0.07					
LP	−0.21	−0.09	−0.07	−0.05	0.02	0.20	0.29	0.31	0.30	0.05	−0.30				
SD	0.24	0.12	0.11	0.09	0.00	−0.16	−0.25	−0.28	−0.37	−0.05	0.34	−0.50			
DTS	0.24	0.12	0.10	0.09	−0.01	−0.18	−0.28	−0.31	−0.39	−0.06	0.36	−0.50	0.99		
Texture	0.03	0.04	−0.18	−0.02	0.10	−0.48	−0.5	−0.48	−0.61	−0.31	0.53	−0.05	0.11	0.14	
DEM	−0.17	−0.05	−0.06	−0.03	0.06	0.16	0.27	0.31	0.28	0.00	−0.34	0.48	−0.54	−0.53	−0.15

Note: LP = landscape position; SD = surface depression; DTS = distance to stream; NDVI = Normalized Difference Vegetation Index; NDSI = Normalized Difference Soil Index; NDWI = Normalized Difference Water Index; DEM = Digital Elevation Model.

**Table 2.** Contrasting the accuracy between three classifiers and combinations of predicator variables. The highest overall accuracy (OA) per test is identified with bold font.

Test	Input Layers	Decision-Tree (DT) Classification					Rule-Based (RB) Classification					Random Forest (RF) Classification			
		Training Data		OA on Testing Data			Training Data		OA on Testing Data			Training Data	OA on Testing Data		
		# Tree Leaves	Error (%)	Mean (%)	95% CI		# “*If- Then*” Rules	Error (%)	Mean (%)	95% CI		Out-of-Box Error (%)	Mean (%)	95% CI	
1	4 traditional bands (B2 + B3 + B5 +B7)	222	6.7	66.9	65.1	68.7	136	7.3	66.5	64.7	68.3	9.9	**73.1**	71.4	74.7
2	5 traditional bands (B1 + B2 + B3 + B5 +B7)	252	5.4	69.2	67.4	70.1	157	6.1	66.6	64.8	68.4	8.8	**74.0**	72.4	75.7
3	8 traditional bands (B1-B8)	270	3.5	73.1	71.4	74.7	168	4.0	74.7	73.0	76.3	6.6	**76.7**	75.1	78.2
4	8 traditional bands + NDVI	255	3.4	73.4	71.7	75.0	161	4.0	73.5	71.8	75.1	6.8	**75.7**	74.0	77.3
5	8 traditional bands + NDWI	251	3.5	72.8	71.1	74.5	152	4.1	73.6	72.0	75.3	6.6	**77.0**	75.4	78.6
6	8 traditional bands + NDSI	219	3.5	73.1	71.4	74.7	165	3.8	71.9	70.2	73.6	6.6	**77.0**	75.4	78.6
7	8 traditional bands + texture	226	2.7	78.2	76.6	79.7	156	3.1	77.7	76.1	79.2	4.9	**81.1**	79.6	82.6
8	8 traditional bands + elevation dataset	139	2.2	61.6	59.8	63.4	162	2.5	61.9	60.0	63.7	4.2	**75.3**	73.6	76.9
9	8 traditional bands + 4 indices (NDVI, NDWI, NDSI, texture)	225	2.4	77.2	75.6	78.8	140	2.9	78.7	77.2	80.2	5.1	**80.6**	79.0	82.0
10	8 traditional bands + 4 spectral indices; with boost (10 trials)	Boost	0.1	80.1	78.6	81.6	Boost	0.0	80.0	78.5	81.5				
11	8 traditional bands + 4 spectral indices + 3 hydrogeomorphology variables	49	0.8	55.3	53.4	57.1	48	0.8	54.8	52.9	56.6	1.6	**74.7**	73.1	76.3
12	8 traditional bands + 4 spectral indices + 3 hydrogeomorphology variables; with boost (10 trials)	Boost	0.0	60.2	58.3	62.0	Boost	0.0	58.9	57.1	60.8				
13	8 traditional bands + 4 spectral indices + 3 hydrogeomorphology variables + elevation dataset	153	0.7	58.0	56.1	59.8	100	0.8	58.3	56.4	60.1	1.3	**73.0**	71.2	74.5
14	8 traditional bands + 4 spectral indices + 3 hydro attributes + elevation dataset; with boost (10 trials)	Boost	0.0	63.1	61.3	64.9	Boost	0.0	59.9	58.0	61.7				
15	Uncorrelated and parsimonious (B1 + B3 + B5 + B7 + texture)	221	3.4	75.7	74.0	77.3	154	3.9	74.0	72.4	75.7	6.2	**81.2**	79.7	82.6
16	Uncorrelated and parsimonious (B1 + B3 + B5 + B7 + texture) with boost	Boost	0.7	80.7	79.2	82.1	Boost	0.7	77.8	76.2	79.3				

**Table 3.** Jeffries-Matusita (J-M) distance measure of class separability for input predictor variables combination of WV2 bands 1–8; the table-wide J-M value was 1.95, indicating high overall separability.

Wetland Class	1	2	3	4	5	6	7	8	9	10	11	12	13	14	15	16	17	18	19	20	21
2	1.95																				
3	2.00	1.96																			
4	2.00	1.99	1.69																		
5	2.00	2.00	2.00	2.00																	
6	2.00	1.86	2.00	2.00	2.00																
7	1.94	1.94	2.00	2.00	2.00	1.98															
8	2.00	1.80	2.00	2.00	2.00	1.54	1.99														
9	2.00	1.66	2.00	2.00	2.00	1.74	2.00	1.12													
10	1.99	1.83	2.00	2.00	2.00	1.86	1.95	1.91	1.89												
11	2.00	1.96	2.00	2.00	2.00	2.00	2.00	1.99	1.97	1.34											
12	2.00	1.99	2.00	2.00	2.00	2.00	2.00	2.00	1.98	1.90	1.64										
13	2.00	1.98	2.00	2.00	2.00	2.00	2.00	1.98	1.92	1.99	1.95	1.73									
14	2.00	1.88	2.00	2.00	2.00	2.00	2.00	1.87	1.52	2.00	2.00	2.00	1.98								
15	2.00	1.95	2.00	2.00	2.00	2.00	2.00	1.99	1.87	2.00	2.00	2.00	2.00	1.57							
16	2.00	1.97	2.00	2.00	2.00	2.00	2.00	2.00	1.89	2.00	2.00	2.00	2.00	1.82	1.70						
17	2.00	1.99	2.00	2.00	2.00	2.00	2.00	2.00	1.98	2.00	2.00	2.00	2.00	1.93	1.98	1.94					
18	2.00	1.98	2.00	2.00	2.00	2.00	2.00	2.00	1.97	2.00	2.00	2.00	2.00	1.98	2.00	1.94	1.70				
19	2.00	2.00	2.00	2.00	2.00	2.00	2.00	2.00	2.00	2.00	2.00	2.00	2.00	1.98	2.00	1.99	1.52	1.69			
20	2.00	1.98	2.00	2.00	2.00	2.00	2.00	2.00	1.93	2.00	2.00	2.00	1.98	1.98	2.00	1.95	1.98	1.79	1.99		
21	2.00	2.00	2.00	2.00	2.00	2.00	2.00	2.00	2.00	2.00	2.00	2.00	2.00	2.00	2.00	2.00	2.00	2.00	2.00	1.68	
22	2.00	1.96	2.00	2.00	2.00	2.00	2.00	1.94	1.74	2.00	2.00	2.00	1.95	1.88	1.95	1.84	1.99	1.95	2.00	1.05	1.71
